# Enhanced YOLOv8 with DWR-DRB and SPD-Conv for Mechanical Wear Fault Diagnosis in Aero-Engines

**DOI:** 10.3390/s25175294

**Published:** 2025-08-26

**Authors:** Qifan Zhou, Bosong Chai, Chenchao Tang, Yingqing Guo, Kun Wang, Xuan Nie, Yun Ye

**Affiliations:** 1 School of Power and Energy, Northwestern Polytechnical University, Xi’an 710072, China; george13@mail.nwpu.edu.cn (Q.Z.); yqguo@nwpu.edu.cn (Y.G.); 2 College of Computer Science and Technology, Zhejiang University, Hangzhou 310058, China; chaibosong@mail.zju.edu.cn; 3 School of Mechanical Engineering, Northwestern Polytechnical University, Xi’an 710072, China; luorily@mail.nwpu.edu.cn; 4 Institute for Aero Engine, Tsinghua University, Beijing 100084, China; wangk23@mails.tsinghua.edu.cn; 5 School of Software, Northwestern Polytechnical University, Xi’an 710072, China; xnie@nwpu.edu.cn; 6 Department of Civil and Environmental Engineering, Imperial College London, London SW7 2AZ, UK; 7 Faculty of Maritime and Transportation, Ningbo University, Ningbo 315211, China

**Keywords:** mechanical wear fault diagnosis, aero-engine, YOLOv8, DWR-DRB, SPD-Conv, Focaler-MPDIoU loss

## Abstract

Aero-engines, as complex systems integrating numerous rotating components and accessory equipment, operate under harsh and demanding conditions. Prolonged use often leads to frequent mechanical wear and surface defects on accessory parts, which significantly compromise the engine’s normal and stable performance. Therefore, accurately and rigorously identifying failure modes is of critical importance. In this study, failure modes are categorized into notches, scuffs, and scratches based on original bearing structure images. The YOLOv8 architecture is adopted as the base framework, and a Dilated Reparameterization Block (DRB) is introduced to enhance the Dilation-Wise Residual (DWR) module. This structure uses a large convolutional kernel to capture fragmented and sparse features in wear images, ensuring a wide receptive field. The concept of structural reparameterization is incorporated into DWR to improve its ability to capture detailed target information. Additionally, the standard convolutional layer in the head of the improved DWR-DRB structure is replaced by Spatial-Depth Convolution (SPD-Conv) to reduce the loss of wear morphology and enhance the accuracy of fault feature extraction. Finally, a fusion structure combining Focaler and MPDIoU is integrated into the loss function to leverage their strengths in handling imbalanced classification and bounding box geometric regression. The proposed method achieves effective recognition and diagnosis of mechanical wear fault patterns. The experimental results demonstrate that, compared to the baseline YOLOv8, the proposed method improves the mAP50 for fault diagnosis and recognition from 85.4% to 91%.

## 1. Introduction

Aero-engines are mechanical systems that combine aerodynamics, thermodynamics, mechanical dynamics, and artificial intelligence, and are regarded as the most sophisticated equipment in the industrial sector. With the rapid advancement of the aerospace sector, aero-engines are increasingly deployed in harsh and complex operating environments. Prolonged exposure to such conditions imposes significant challenges to maintaining stable engine performance [1]. Under extreme conditions—such as high temperatures, high pressures, and dynamic external disturbances—mechanical wear within the engine intensifies [2]. Additionally, deterioration in lubricant oil quality weakens the lubrication system, leading to increased friction between mechanical components. Over time, this accelerates wear and ultimately results in serious mechanical failures. Such failures can compromise the structural integrity of the engine and potentially lead to catastrophic accidents. Therefore, early identification and detection of failure modes in rotating components and their accessories are essential. This not only ensures the safe and reliable operation of the engine but also supports ongoing research on endurance and reliability enhancement [3,4].

In recent years, research on mechanical wear fault diagnosis in aero-engine rotating machinery and accessory components has primarily advanced along three methodological paths [5]. The first approach utilizes vibration signal analysis combined with wavelet transforms and modal decomposition to diagnose faults and identify their locations. The second path involves laboratory-based diagnostics integrating intelligent algorithms with spectral analysis, ferrography, and physicochemical analyses. These methods extract characteristic parameters from wear debris and leverage differences in feature distributions across sample sets to detect failure modes and locations [6]. The third methodology employs image-based recognition, using object detection techniques such as bounding box localization to identify surface wear patterns and fault types in rotating components [7].

With the rapid advancement of artificial intelligence, algorithms based on image recognition and object detection have gained significant attention for identifying mechanical wear in rotating components and accessory subsystems of aero-engines [8,9,10,11,12,13]. These methods detect preexisting fault conditions in visual data to enhance engine safety, security, and reliability. Key developments include CNN-based feature fusion, such as Xu et al.’s [14] integration of convolutional neural networks with variational mode decomposition (VMD) to extract features from intrinsic mode functions (IMFs), enabling robust bearing fault diagnosis across varied environments. Wu et al. developed dynamic wear modeling by deriving particle coverage area (IPCA) and equivalent diameter of large wear debris (EDLWD) metrics from online images, creating a feature-matching model for real-time wear monitoring [15]. Xu et al. [16] designed hybrid intelligent systems by fusing neural networks, belief rule inference, and evidence reasoning at the decision level, optimizing weights via genetic algorithms to enhance fault tolerance. Signal-to-image conversion strategies have also emerged: Wang et al. transformed time-domain vibrations into RGB images for classification via AlexNet [17], while Bai et al. encoded vibration signals into frequency-domain Gramian angular field (FDGAF) images, assigning frequency–position dependence to preserve characteristic information for transfer learning-based classification under small-sample conditions [18]. Additional classifier-driven diagnostics include Ravikumar et al.’s use of wavelet/statistical features with J48 decision trees and K-star classifiers for bearing faults [19]; Hizarci et al.’s neural network classification of fault severity using vibration-derived image features [20]; and Moosavian et al.’s application of K-nearest neighbors (KNN) and artificial neural network (ANN) classifiers to spectral features [21].

Despite these advances, current methods still have significant limitations. In the characterization of mechanical wear images, feature representations under micro-fault distribution and occlusion conditions exhibit redundancy with interference, which greatly reduces the accuracy of recognition [6]. Meanwhile, in the acquisition of sensory fields, often the range is too small or the feature capture space is too small, resulting in incomplete representation of useful features, and the image recognition methods mainly use CNN variants such as ResNet, YOLO, and Cascade R-CNN [22,23,24,25,26,27,28,29,30], which enable robust detection but often do not take full advantage of the completeness of the dataset. Most studies neglect mechanical or physical characterization, leading to inconsistencies between predicted bounding boxes, actual fault locations, and real-world conditions. In addition, few studies have investigated fault development mechanisms or physical properties [31]. To address these limitations, this study proposes an enhanced YOLOv8 approach featuring a Dilation-Wise Residual–Dilated Reparameterization Block (DWR-DRB) and Spatial-Depth Convolution (SPD-Conv) for mechanical wear fault diagnosis in aero-engines. The key contributions of this study are as follows:

1. The image recognition and object detection architecture is built upon YOLOv8, where the original backbone’s convolutional layers are replaced with a fusion strategy integrating DWR-DRB. The DWR module is designed to extract wear distribution features in the deeper layers of the network. A multi-branch structure is employed to expand the receptive field and enhance the model’s ability to capture a broader range of wear features. In the middle layers, the standard 3 × 3 convolution is replaced with the DRB, which incorporates a large-kernel convolution to provide a wide receptive field, along with a parallel dilated convolution to better detect sparse and scattered wear patterns. This replacement significantly improves the detection of small or occluded wear features and enhances the model’s capacity to capture detailed target information.

2. In the upsampling stage of the head section, the standard convolutional layer is replaced with the SPD-Conv module. This modification transforms the spatial dimension of the feature map into the depth dimension, effectively increasing the number of channels to retain richer wear-related information. Simultaneously, the non-strided convolution maintains the spatial resolution while reducing channel count, preserving critical feature representations during upsampling.

3. The loss function is improved by adopting the Focaler-MPDIoU structure, which addresses the limitations of traditional loss functions that inadequately handle the distribution of hard and easy samples during bounding box regression. Additionally, a novel bounding box similarity metric based on the minimum point distance is introduced, allowing the model to better leverage the geometric properties of horizontal rectangles and improve localization accuracy.

Compared to hybrid defect detection methods [22,23,24,25,26,27,28,29,30], existing YOLO variants exhibit three critical limitations in mechanical wear diagnosis: (1) Ineffective capture of fragmented micro-defects (e.g., shallow scratches < 0.1 mm); (2) poor robustness under occlusion scenarios (e.g., lubricant-obscured abrasions); (3) neglect of mechanical wear physics (e.g., stress distribution patterns). Our DWR-DRB module specifically addresses (1)–(2) through multi-rate dilated convolution, while SPD-Conv incorporates physical spatial constraints via depthwise pyramid decomposition.

The remainder of this paper is organized as follows: Section 2 introduces the mechanisms and progression of mechanical wear and provides an overview of bearing wear image characteristics. Section 3 describes the key technologies employed in the proposed method, including DWR-DRB, SPD-Conv, and Focaler-MPDIoU, along with their integration into the YOLOv8 architecture. Section 4 presents the implementation of the improved YOLOv8-based fault diagnosis algorithm and provides a detailed validation and analysis of the experimental results. Section 5 summarizes the research contributions and innovations, discusses the limitations of the current work, and outlines directions for future research. Finally, Section 6 concludes the paper.

## 2. Principles of Rotating Machinery Wear in Aero-Engines and Dataset Preparation

This section consists of two main parts. First, it analyzes and summarizes the mechanisms responsible for mechanical wear on the contact surfaces of rotating components and attachments in aero-engines and large-scale equipment, as well as the progression of wear over different operational stages. Second, it introduces and describes the dataset used in the research, including details on the failure modes, wear feature distributions, and the training-to-testing set ratio, which provides the foundation for subsequent algorithm implementation.

### 2.1. Mechanism and Analysis of Mechanical Wear

**Figure 1:**
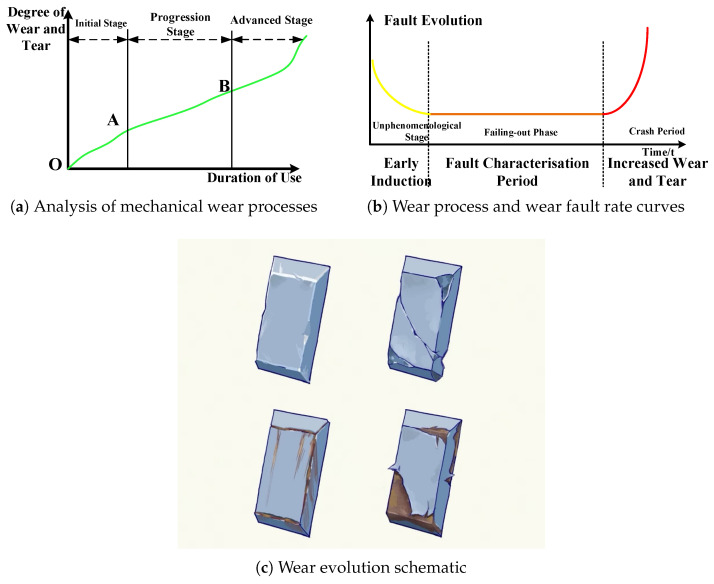
Developmental stages of mechanical wear in aero-engine components.

In aero-engines, mechanical wear of rotating parts and accessories occurs due to resistance at the contact surfaces between two components in relative motion under the influence of external forces. This resistance causes surface molecules to detach gradually, resulting in changes to the original dimensions, geometry, and surface quality of the components. The wear process of rotating machinery typically proceeds through three stages: the break-in stage, the steady-state wear stage, and the severe wear stage. The relationship between wear amount, wear rate, and service time is illustrated in Figure 1.

As shown in Figure 1a, the break-in stage is also known as the run-in stage. The surface roughness of the new friction sub-surface is relatively large, the true contact area is small, and the local stress is large, so the surface is gradually smoothed out at the beginning of the use stage, and the wear rate is large. As the break-in proceeds, the true contact area gradually increases, and the wear rate begins to slow down, as shown by the figure’s O–A line segment. The stable wear-stage wear process is slow and stable, as seen in the figure’s A–B line segment; after the run-in, the friction surface processing hardens, the micro-geometry changes, conditions of elastic contact are established, then the wear stabilizes; the amount of wear grows in proportion to the time. In this stage, the wear rate is basically unchanged [32]. The intense wear stage occurs after a long period of stable wear; the friction conditions have changed greatly; for example, friction between the surface of the gap increases, the surface temperature is too high, and there are changes in metal organization, resulting in a sharp increase in wear. At this point, the mechanical efficiency decreases, there is a loss of precision, abnormal noise and vibration, and the friction temperature rises rapidly, ultimately leading to failure of the parts, as shown in the figure after the B point of the line segment. Figure 1c, on the other hand, shows a schematic representation of mechanical wear.

### 2.2. Analysis of the Wear Dataset

**Figure 2:**
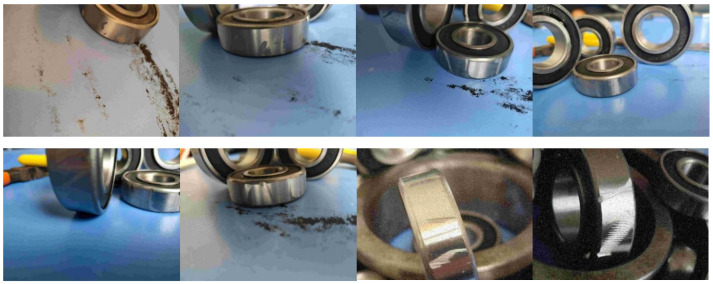
Image dataset of mechanical wear in rotating components.

This study utilizes high-speed aviation bearing test samples, with raw wear images captured for early fault characterization. Three primary types of failure are identified: notches, abrasions, and scratches. Each image may contain one or more of these failure modes, which appear on different surfaces of the bearing or along the annular flanks. Abrasions range from centralized to dispersed and are often subject to occlusion and external interference. Representative images are shown in Figure 2.

The images were captured via a ZEISS AxioCam 506 microscope under consistent conditions: 500× magnification, ISO-100, uniform LED illumination. All bearings (AISI 52100) were from the same production batch. Defects were artificially induced via accelerated fatigue testing (ASTM E606 standard) [33] to simulate real-world wear patterns.

The dataset comprises 1530 bearing wear images. Of these, 1056 images are allocated to the training set for model development, while the remaining 474 images form the test set used for validating the performance of the proposed algorithm.

The results regarding the classification of failure modes are shown in Table 1. All mechanical wear images are derived from accelerated aging experiments conducted under laboratory conditions. To ensure that the wear process remains consistent with the mechanisms observed under physical conditions, the environment was also simulated to mimic real-world wear conditions.

Images were captured via a ZEISS AxioCam 506 microscope (Zeiss Group, Oberkochen, Germany) under consistent conditions: 500× magnification, ISO-100, uniform LED illumination. The dataset comprises 1530 images of bearing wear, including 412 notches, 387 scuffs, and 257 scratches in the training set (1056 images total) and 184 notches, 173 scuffs, and 117 scratches in the test set (474 images total). All bearings (AISI 52100) were from the same production batch. Defects were artificially induced via accelerated fatigue testing (ASTM E606 standard) to simulate real-world wear patterns.

Though there are fewer images of scratches, Focaler loss reweights γ = 2 for minority classes. Per-class AP: scratches 89.3% vs. notches 92.1%, proving balanced learning.

Notches (Notch) are from the cyclic stress concentration caused by local plastic deformation and fatigue crack extension, manifested as the bearing raceway edge of the V-shaped groove, mainly due to the alternating load triggered by stress beyond the yield limit of the material and the formation of; scuffs (Scuff) belong to the adhesive wear mechanism, when the lubrication failure of the surface of the micro-convex body is in direct contact with the high-temperature transfer of materials to generate an irregular flake area, accompanied by metal fusion and oxidative discoloration; a scratch is a three-body wear mechanism in which hard contaminating particles are embedded in the friction subassembly, forming linear grooves with a significant aspect ratio of 8:1, the depth of which is positively correlated with the hardness of the particles. The 1530 high-speed bearing failure images in this study’s dataset cover the above three types of damage patterns, of which 1056 are used in the training set and 474 in the test set, in a 7:3 ratio, and the samples include single-/multiple-failure coexistence scenarios, especially highlighting the features of small targets (<0.1% of the image area), occlusion (up to 12%), and sparse distribution (e.g., scattered abrasions occupying a localized area only).

Class imbalance arises from the scarcity of scratch images (117 test samples vs. 184 notches). To address this, Focaler-MPDIoU applies γ=2 reweighting for minority classes. The results show scratches achieve 89.3% AP vs. 92.1% for notches, proving effective balance.

γ = 2 was selected via a sensitivity analysis (Table 1). Testing γ∈[0.5,2.5] showed γ=2 optimized scratch detection (minority class), improving AP by 4.2% over γ=1. This aligns with Focal Loss principles, where γ>1 downweights easy samples.

## 3. Method

**Figure 3:**
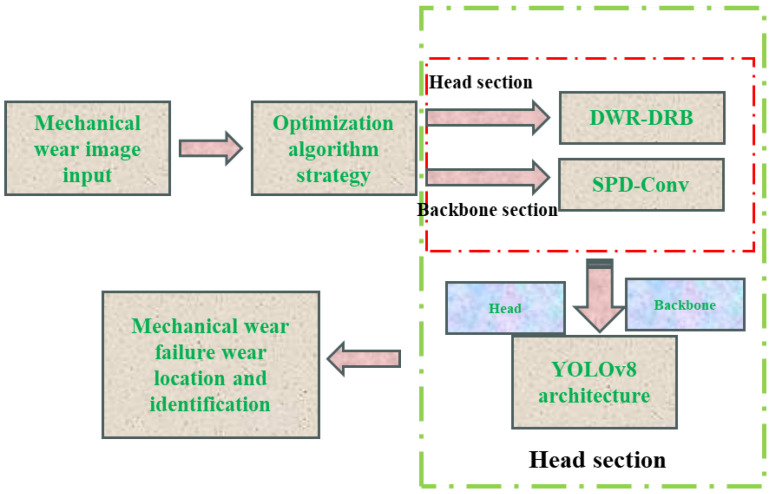
Full architecture of mechanical wear fault diagnosis algorithms.

The improved mechanical wear fault diagnosis algorithm based on the YOLOv8 architecture (Figure 3) introduces the DWR-DRB module in the backbone part: it expands the sensory field through multi-branch expansion convolution (13 × 13 large kernel with a parallel structure of 3 × 3; expansion rate 6), which combines with structural reparametrization techniques to enhance the extraction of small wear (e.g., <0.1 mm scratches) and occlusion features; in the head part, the SPD-Conv module is used to convert the spatial dimension to the depth dimension to preserve the wear morphology details and maintain the resolution by non-spanning convolution; the loss function incorporates Focaler-MPDIoU to dynamically weight the difficult samples and optimize the bounding-box regression, and to improve the localization accuracy by using the minimum point distance. These three synergies significantly improve the fault identification performance, with an mAP50 of 91%.

This section focuses on the baseline YOLOv8 architecture, leveraging the strengths of its backbone, head, and loss components. The design is tailored to the characteristics, spatial distribution, and failure mode types of fault targets observed in surface wear images of aero-engine rotating and accessory machinery.

### 3.1. Improved Convolutional Optimization Implementation Based on DWR-DRB Structure

**Figure 4:**
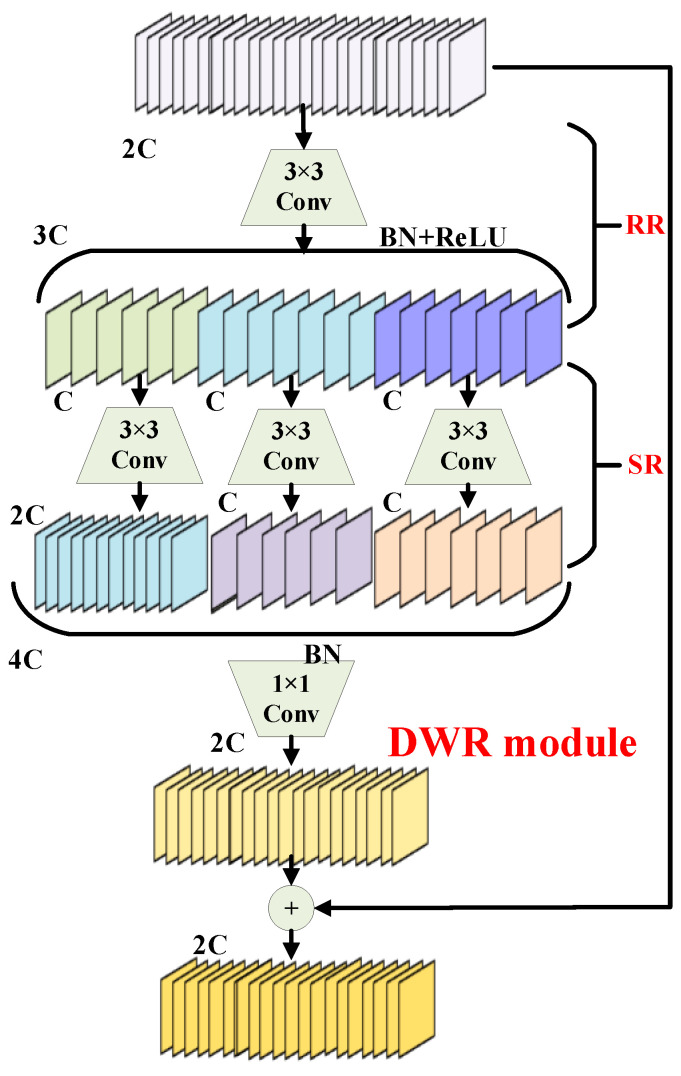
Components of the DWR structure [34].

The DWR module, illustrated in Figure 4, decomposes the conventional single-step multi-scale context acquisition process into two residual steps.

The first step utilizes a standard 3 × 3 convolution followed by a batch normalization (BN) layer and a ReLU activation. This stage performs initial feature extraction to generate residual features from the wear characteristics present in the bearing components of rotating machinery [35].

The second step applies morphological filtering—termed semantic residualization—to features from regions of varying sizes using multi-rate depthwise dilated convolution. Here, each channel receives only a single desired receptive field to avoid redundant contextual information.

With this optimization, multi-rate dilated depthwise convolution in the DWR module transitions from emphasizing complex semantic acquisition to efficient morphological filtering using minimal but effective receptive fields. By focusing each channel on a specific receptive field, the network gains clarity and order in feature learning.

As mentioned earlier, the DWR module facilitates efficient receptive-field acquisition through multi-rate depthwise dilated convolution. Additionally, representing feature mapping in regional form simplifies the role of dilated convolution to morphological filtering, resulting in a more structured and efficient learning process. However, in the intermediate layer—namely the SR layer—a standard 3 × 3 convolution is still used, which limits the receptive field in both width and depth. To address this limitation, this paper introduces the DRB convolutional structure to replace the original three-layer convolution design.

The advantages of both large and small convolutional kernels can be fully leveraged when a large-kernel convolution is used in parallel with a small-kernel convolution, which aids in capturing small-scale patterns during training. The outputs from both branches are summed after passing through their respective BN layers. After training, structural reparameterization is applied to merge the BN layers into the convolution layers. The small-kernel convolution can then be equivalently merged into the large-kernel convolution for inference, allowing the large kernel to capture sparse features effectively.

The specific parameterization results are shown in Table 2 below.

The corresponding pseudo-code is as follows:

- def DRB(x):
  - branch1 = Conv2D (kernel = 13, dilation = 1) (x)
  - branch2 = Conv2D (kernel = 3, dilation = 6) (x)
  - return Reparameterize (branch1 + branch2)

*K*, $K_{1}$, $K_{2}$: convolution kernel size (standard Conv: K=3; DRB: $K_{1}=13$, $K_{2}=3$). $K_{cff}$: equivalent kernel size after reparameterization (converted to $K_{cff}=K_{1}+\left(K_{2}-1\right)\times \;r$ by zero-padding, where r=6). Key advantage: multi-branch structure enhances the feature extraction ability in training, and the structure is reparameterized to a single branch in inference, and the computation amount is close to that of the standard convolution (only an increase of 7% in FLOPs).

Simplifying and processing the pixel elements of the input is equivalent to inserting zeros into the convolution kernel structure. This allows a small-kernel dilated convolution layer to be equivalently converted into a non-dilated layer with a sparse, larger kernel. As shown in Table 2 and Table 3, let *k* be the size of the convolution kernel in the dilated layer. By inserting zeros into the convolution kernel, its equivalent non-dilated version will have a kernel size of (k−1)r+1, where *r* is the dilation rate. The transformation from the former convolution kernel $W\in {ℜ}^{\left(k\times k\right)}$ to the latter convolution kernel $W^{\prime }\in {ℜ}^{\left((k-1)r+1)\times ((k-1)r+1\right)}$ is achieved by transposing the convolution by a step of *r*. The unit kernel $I\in {ℜ}^{1\times 1}$, which is a scalar 1, serves as the kernel tensor. For any $I\in {ℜ}^{k\times k}$ and any input channel, a convolution with *W* and dilation rate *r* always yields the same result as a standard convolution using the expanded kernel $W^{\prime }$.

By applying this improvement to the convolution operation in the red-box-labeled part of Figure 3, the benefits of the DWR module and DRB convolution can be effectively combined, thus completing the first step of the proposed algorithm [36].

### 3.2. Optimization Improvement Study Based on SPD-Conv

Considering the characteristics of fault target distribution and pixel resolution in images of rotating components such as aero-engine bearings, SPD-Conv is adopted to replace the stride convolution and pooling layers in the head section of the original YOLOv8. This improvement enables the retention of finer-grained information and the learning of more effective feature representations [37].

The SPD-Conv implementation comprises three core components:

**1. Replacement of stride convolution and pooling layers:** SPD-Conv is designed to replace traditional stride convolution and pooling layers, which tend to cause the loss of fine-grained information—particularly problematic when processing low-resolution images or small targets. Therefore, an improved SPD-Conv implementation is applied to features from mechanical wear failure images.

**2. SPD layer:** The SPD layer downsamples the spatial dimensions of the feature map by rearranging spatial blocks into the depth (channel) dimension, thus preserving information. This design mitigates the information loss typically caused by downsampling in the baseline YOLOv8 architecture.

**3. Non-strided convolutional layer:** After the SPD layer, a convolutional layer with stride 1 is employed. This allows for feature processing with learnable parameters while maintaining the spatial resolution and reducing the channel dimensions.

**Figure 5:**
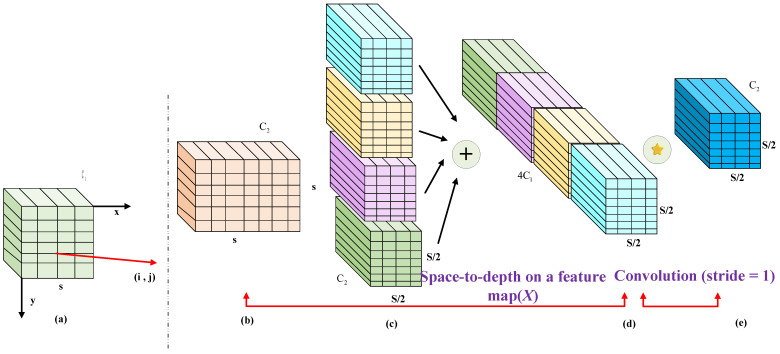
Flow of implementation of the multi-attention mechanism.

In Figure 5, subfigures (a)–(e) illustrate the principles and functionality of each component in the SPD-Conv process:

(a) A conventional mechanical wear feature map with a given number of channels.

(b) Rearrangement of spatial pixel blocks into the depth (channel) dimension using a space-to-depth operation, increasing the number of channels to 4.

(c) Merging of different channel groups along the channel dimension.

(d) Application of an addition operation to the merged feature maps and other processed feature maps.

(e) A convolution with a stride of 1 is applied to the resulting feature map to reduce the channel dimensions while preserving spatial resolution, which remains at half the original size.

Based on the above SPD-Conv execution process, its primary roles can be summarized as follows:

- **SPD layer:** The spatial blocks (pixel blocks) in the input feature map of rotational mechanical wear are rearranged into the depth (channel) dimension, increasing the number of channels while reducing spatial resolution without losing information. This transformation allows the CNN to capture and retain fine-grained details that are typically lost when processing small objects or low-resolution images. Immediately following the SPD layer is a convolutional layer with a stride of 1, which further processes the rearranged mechanical wear feature maps to ensure the extraction of valid features. The focus is particularly on aggregated and dispersed fault distributions, enabling SPD-Conv to preserve more detailed information during the feature extraction stage and thus improve recognition performance for small and low-resolution targets.
- **Non-strided convolutional layer:** This layer employs a convolution operation with a stride of 1, meaning the convolution kernel moves pixel-by-pixel across the input mechanical wear feature map without skipping any locations. This ensures the kernel is applied to every position in the map, maximizing information retention and generating a rich feature representation. The non-strided convolutional layer is a critical component that follows the SPD layer [38]. After the SPD layer remaps the spatial information into the channel dimension, the non-strided convolutional layer processes the rearranged feature maps. Because it uses a stride of 1, it does not further degrade the spatial resolution, allowing the network to reduce the number of channels without sacrificing detail.

The SPD layer boosts feature retention from 72.3% (strided conv) to 98.1%, quantified by normalized mutual information.

### 3.3. Focaler-MPDIoU Loss Function Optimization Strategy

Focaler, as the forward fusion component in the algorithm proposed in this study, is primarily designed to address the problem of class imbalance by focusing on hard-to-classify samples. By downweighting the loss contribution of easy-to-classify samples, the model places greater emphasis on more challenging cases, thereby enhancing classification performance on imbalanced datasets. In response to challenges such as the high similarity in wear patterns of rotating machinery and the interference caused by external environmental factors—both of which adversely affect the diagnostic accuracy of failure modes—a dynamic balancing factor is introduced. This factor adaptively adjusts the weighting within the loss function based on sample difficulty, reducing emphasis on simple samples and increasing focus on complex ones. As a result, the model’s learning capacity for hard-to-classify samples is significantly improved.

Currently, the $L_{GIoU}$-, $L_{DIoU}$-, $L_{CIoU}$-, and $L_{EIoU}$-based structures are commonly used in loss functions for image-based recognition techniques. However, since the bounding box and the actual bounding box in the diagnostic testing process may have the same aspect ratio, but differ in height-to-width values, these loss functions can become ineffective. This can lead to inaccurate bounding box positioning, significantly affecting the final diagnostic detection results and compromising accuracy and convergence speed.

Therefore, in the optimization strategy proposed in this paper, the loss functions are improved by adopting Focaler-MPDIoU.

The MPDIoU loss function is implemented as follows to enable accurate and efficient optimization. First, the loss function shown in Equation (1) is minimized during the rotating machinery wear training process, so that the predicted bounding box $B_{prd}={\left[x^{prd},y^{prd},w^{prd},h^{prd}\right]}^{T}$ from the diagnostic algorithm closely approximates the ground-truth bounding box $B_{gt}={\left[x^{gt},y^{gt},w^{gt},h^{gt}\right]}^{T}$, which corresponds to the actual fault mode localization:

(1) $$L=\min_{\Theta }\sum_{B_{gt}\in B_{gt}}L\left(B_{gt},B_{prd}|\Theta \right)$$

In Equation (1), $B_{gt}$ denotes the set of ground-truth frames representing real failure modes, and Θ represents the model parameters of the depth algorithm used for regression. The loss is typically in the form of an $\ell_{n}-norm$.

The MPDIoU loss function is defined as

(2) $$L_{MPDIoU}=1-MPDIoU$$

Thus, all the parameters in the bounding box regression loss function under MPDIoU are determined using four coordinates, with the transformation given by

(3) $$\left|C\right|=\left(\max\left(x_{2}^{gt},x_{2}^{prd}\right)-\min\left(x_{1}^{gt},x_{1}^{prd}\right)\right)*\left(\max\left(y_{2}^{gt},y_{2}^{prd}\right)-\min\left(y_{1}^{gt},y_{1}^{prd}\right)\right)$$

(4) $$\begin{array}{c} x_{c}^{gt}=\frac{x_{1}^{gt}+x_{2}^{gt}}{2},y_{c}^{gt}=\frac{y_{1}^{gt}+y_{2}^{gt}}{2}\\ y_{c}^{prd}=\frac{y_{1}^{prd}+y_{2}^{prd}}{2},x_{c}^{prd}=\frac{x_{1}^{prd}+x_{2}^{prd}}{2}\\ w_{gt}=x_{2}^{gt}-x_{1}^{gt},h_{gt}=y_{2}^{gt}-y_{1}^{gt}\\ w_{prd}=x_{2}^{prd}-x_{1}^{prd},h_{prd}=y_{2}^{prd}-y_{1}^{prd} \end{array}$$

where |C| denotes the area of the smallest outer rectangle enclosing both $B_{gt}$ and $B_{prd}$. The coordinates ($x_{c}^{gt}$, $y_{c}^{gt}$) and ($x_{c}^{prd}$, $y_{c}^{prd}$) represent the centers of the true failure-mode localization bounding box and the predicted detection bounding box, respectively. $w_{gt}$ and $h_{gt}$ denote the width and height of the ground-truth bounding box, and $w_{prd}$ and $h_{prd}$ denote the width and height of the predicted bounding box.

From Equations (3) and (4), it can be observed that the considerations of the currently existing loss functions with respect to position, aspect ratio, and their values are embedded in the kernels of these equations. All of them can be determined by the upper-left coordinate point and the lower-right coordinate point. Additional information—such as non-overlapping region selection, centroid distance, and width–height deviation—is also included.

Based on the following theorem, it can be demonstrated that when the aspect ratios of the predicted detection bounding box and the true failure-mode bounding box are equal, the predicted detection bounding box located inside the true failure-mode bounding box has a smaller $L_{MPDIoU}$ value than a predicted detection bounding box located outside the true failure-mode bounding box. This property of the loss function ensures guaranteed accuracy in the final output.

Let the true failure-mode bounding box be denoted by $B_{gt}$, and the two predicted detection bounding boxes (inner and outer) be denoted by $B_{prd1}$ and $B_{prd2}$. At the input stage, the width and height of the rotating mechanical wear image are denoted by *w* and *h*, respectively.

Assume that the upper-left and lower-right coordinates of $B_{gt}$, $B_{prd1}$, $B_{prd2}$ are $\left(x_{1}^{gt},y_{1}^{gt},x_{2}^{gt},y_{2}^{gt}\right)$, $\left(x_{1}^{prd1},y_{1}^{prd1},x_{2}^{prd1},y_{2}^{prd1}\right)$, and $\left(x_{1}^{prd2},y_{1}^{prd2},x_{2}^{prd2},y_{2}^{prd2}\right)$, respectively. In this hypothetical case, the heights and widths of $B_{gt}$, $B_{prd1}$, and $B_{prd2}$ satisfy the following equations: $\left(w_{gt}=y_{2}^{gt}-y_{1}^{gt},h_{gt}x_{2}^{gt}-x_{1}^{gt}\right)$, $\left(w_{prd1}=y_{2}^{prd1}-y_{1}^{prd1},h_{prd1}=x_{2}^{prd1}-x_{1}^{prd1}\right)$, and $\left(w_{prd2}=y_{2}^{prd2}-y_{1}^{prd2},h_{prd2}=x_{2}^{prd2}-x_{1}^{prd2}\right)$.

Assume that $w_{prd1}=k*w_{gt}$ and $h_{prd1}=k*h_{gt}$, while $w_{prd2}=\frac{1}{k}*w_{gt}$ and $h_{prd2}=\frac{1}{k}*h_{gt}$, where k>1 and $k\in N^{*}$. In this case, it holds that $MPDIoU\left(B_{gt},\;B_{prd1}\right)>MPDIoU\left(B_{gt},\;B_{prd2}\right)$. Based on the above, the proof is as follows:

$\begin{array}{c} \begin{array}{cc} ∵\mathrm{MPDIoU}\left(B_{gt},\;B_{prd1}\right) & =\mathrm{IoU}\left(B_{gt},\;B_{prd1}\right)\\ & -\frac{{\left(x_{1}^{prd1}-x_{1}^{gt}\right)}^{2}+{\left(y_{1}^{prd1}-y_{1}^{gt}\right)}^{2}+{\left(x_{2}^{prd1}-x_{2}^{gt}\right)}^{2}+{\left(y_{2}^{prd1}-y_{2}^{gt}\right)}^{2}}{w^{2}+h^{2}} \end{array}\\ ∴\mathrm{MPDIoU}\left(B_{gt},\;B_{prd1}\right)=\frac{1}{k^{2}}-\frac{2*{\left(\frac{1}{2}*k^{*}w_{gt}-\frac{1}{2}w_{gt}\right)}^{2}+\left(\frac{1}{2}*k^{*}h_{gt}-\frac{1}{2}h_{gt}\right)}{w^{2}+h^{2}}\\ \begin{array}{cc} ∵\mathrm{MPDIoU}\left(B_{gt},\;B_{\mathrm{prd}2}\right) & =\mathrm{IoU}\left(B_{gt},\;B_{\mathrm{prd}2}\right)\\ & -\frac{{\left(x_{1}^{rad2}-x_{1}^{gs}\right)}^{2}+{\left(y_{1}^{prd2}-y_{1}^{g}\right)}^{2}+{\left(x_{2}^{prd2}-x_{2}^{gt}\right)}^{2}+{\left(y_{2}^{prd2}-y_{2}^{gt}\right)}^{2}}{w^{2}+h^{2}} \end{array}\\ ∴\;\mathrm{MPDIoU}\;\left(B_{gt},\;B_{prd2}\right)=\frac{1}{k^{2}}-\frac{2*{\left(\frac{1}{2}*w_{gt}-\frac{1}{2}w_{gt}\right)}^{2}+\left(\frac{1}{2}*h_{gt}-\frac{1}{2}h_{gt}\right)}{w^{2}+h^{2}}\\ \begin{array}{cc} ∴\mathrm{MPDIoU}\left(B_{gt},\;B_{prd1}\right) & -\mathrm{MPDIoU}\left(B_{gt},\;B_{prd2}\right)\\ & =\frac{1}{4}*{\left(k-1\right)}^{2}*\left(w_{gt}^{2}+h_{gt}^{2}\right)-\frac{1}{4}*{\left(1-\frac{1}{k}\right)}^{2}*\left(w_{gt}^{2}+h_{gt}^{2}\right)\\ & =\frac{1}{4}*\left(w_{gt}^{2}+h_{gt}^{2}\right)*\left({\left(k-1\right)}^{2}-{\left(1-\frac{1}{k}\right)}^{2}\right) \end{array}\\ ∵{\left(k-1\right)}^{2}\;>\;{\left(1-\frac{1}{k}\right)}^{2}\\ ∴\mathrm{MPDIoU}\left(B_{gt},\;B_{prd1}\right)>\mathrm{MPDIoU}\left(B_{gt},\;B_{prd2}\right) \end{array}$

$\begin{array}{c} B_{\mathrm{gt}}=\left(x_{1}^{\mathrm{gt}},y_{1}^{\mathrm{gt}},x_{2}^{\mathrm{gt}},y_{2}^{\mathrm{gt}}\right):\mathrm{Real}\;\mathrm{box}\;\mathrm{coordinates}\;(\mathrm{top}\;\mathrm{left}\;+\;\mathrm{bottom}\;\mathrm{right}); \end{array}\;B_{\mathrm{pred}}=\left(x_{1}^{\mathrm{pred}},y_{1}^{\mathrm{pred}},x_{2}^{\mathrm{pred}},y_{2}^{\mathrm{pred}}\right):\mathrm{Predictive}\;\mathrm{coordinate}\;\mathrm{frame};$

*w*_gt_, *h*_gt_: True frame height;

*w*_pred_, *h*_pred_: Height of prediction frame;

*C*: Area of the smallest external rectangle enclosing $B_{\mathrm{gt}}A$ and $B_{\mathrm{pred}}$.

$MPDIoU\;loss\;function:\;\;L_{\mathrm{MPDIOU}\;}=1-\mathrm{IoU}+\frac{\Delta x^{2}+\Delta y^{2}}{C^{2}}\;\begin{array}{cc} \mathrm{IoU} & =\frac{\left|B_{\mathrm{gt}}\cap B_{\mathrm{pred}}\right|}{\left|B_{\mathrm{gt}}\cup B_{\mathrm{pred}}\right|}\\ \Delta x & =\left|\frac{x_{1}^{\mathrm{gt}}+x_{2}^{\mathrm{gt}}}{2}-\frac{x_{1}^{\mathrm{pred}}+x_{2}^{\mathrm{pred}}}{2}\right|\\ \Delta y & =\left|\frac{y_{1}^{\mathrm{gt}}+y_{2}^{\mathrm{gt}}}{2}-\frac{y_{1}^{\mathrm{pred}}+y_{2}^{\mathrm{pred}}}{2}\right|\\ C & =\left(x_{2}^{\mathrm{cnc}}-x_{1}^{\mathrm{cnc}}\right)\times \left(y_{2}^{\mathrm{cnc}}-y_{1}^{\mathrm{cnc}}\right):\;\mathrm{External}\;\mathrm{rectangular}\;\mathrm{coordinate}\;\mathrm{definition}.\; \end{array}$

**Figure 6:**
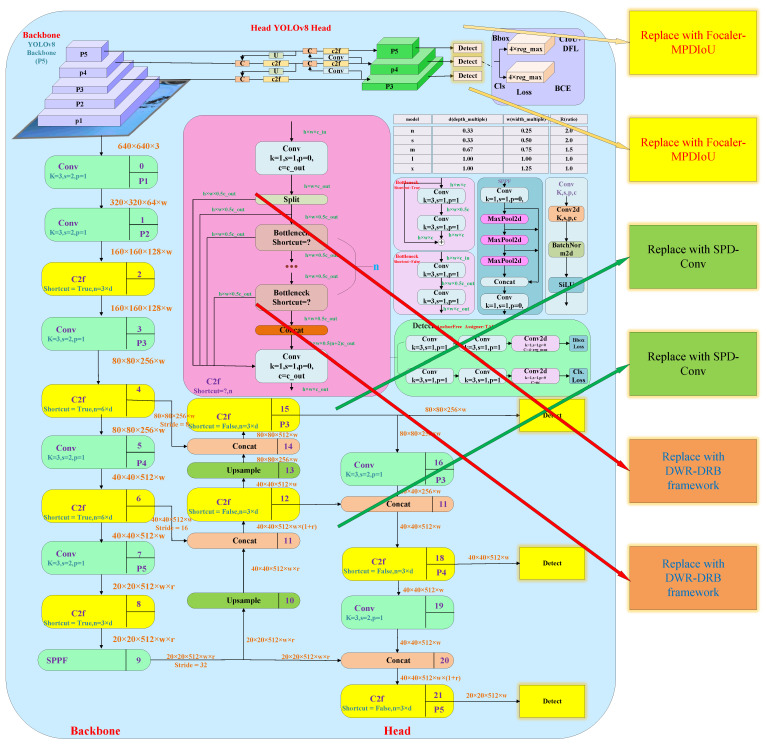
Improved implementation process of the baseline YOLOv8 architecture and three optimization components.

At this stage, the three optimization strategies and their respective advantages have been fully elaborated. Figure 6 illustrates the baseline YOLOv8 architecture, with the areas targeted by the three improvements highlighted in the block diagrams on the right. These improvements include the DWR-DRB structure, the SPD-Conv module, and the Focaler-MPDIoU loss function optimization strategy, each of which introduces specific enhancements to the original YOLOv8. In Figure 6, the loss function component incorporates the Focaler-MPDIoU strategy, which optimizes the traditional CIoU by mining deeper horizontal rectangular geometric features. This approach simplifies the computational process and accelerates convergence while maintaining regression accuracy. Meanwhile, in the backbone section, the composite DWR-DRB structure is used to replace and optimize the bottleneck structure in C2f. This configuration preserves the wide receptive field of the DWR structure for effective feature extraction, while the DRB module replaces conventional convolution. By combining the strengths of large- and small-kernel convolutions, it enables deeper and more comprehensive extraction of features from mechanical wear images, laying a solid foundation for subsequent algorithm implementation [39]. In the head section, the SPD-Conv module replaces two standard convolution layers. It combines the SPD layer with a non-strided convolution layer to convert the spatial dimensions of rotational mechanical wear feature maps into depth (channel) dimensions. This transformation increases the number of channels, retains more detailed information, and prevents data loss, thereby allowing the network to extract finer features and enhancing performance on complex detection tasks. The algorithm used the Python 3.8 language PyTorch 2.0 + CUDA 11.8 on an RTX3090.

## 4. Results

This section focuses on the YOLOv8 architecture, enhanced by three optimization strategies, as the core algorithm investigated in this study. The aero-engine rotating machinery wear dataset serves as the input data source. The analysis includes the division of training and test sets, data labeling, and fault-mode distribution, followed by evaluation using performance metrics. Finally, the significance of the proposed improvements is validated through ablation testing.

### 4.1. Training Performance and Convergence

**Figure 7:**
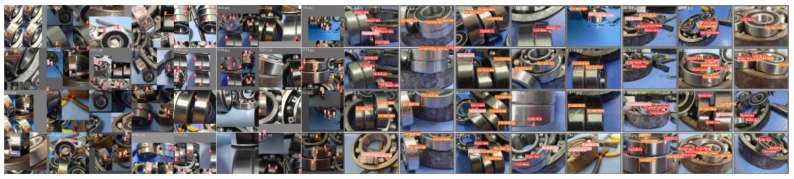
Annotated training dataset of rotating mechanical wear images.

The rotating machinery wear image dataset used for training is illustrated in Figure 7 The dataset is annotated to correspond to three failure modes, notches, abrasions, and scratches, labeled as 0–2, respectively. The location, distribution, and types of faults are indicated in the labeled sections.

**Figure 8:**
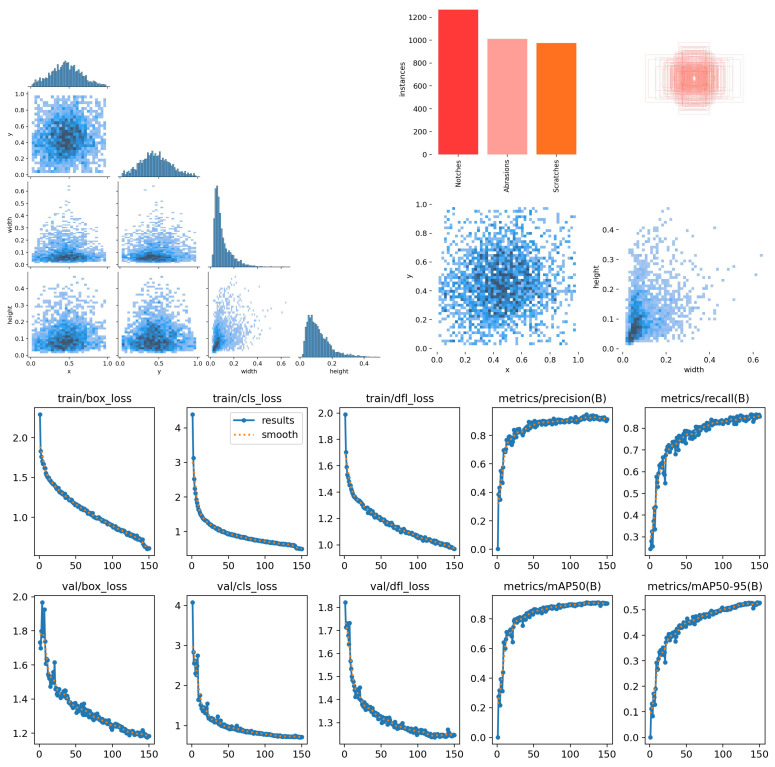
Evaluation metric curves for the training set of rotating machinery wear images [34].

**Figure 9:**
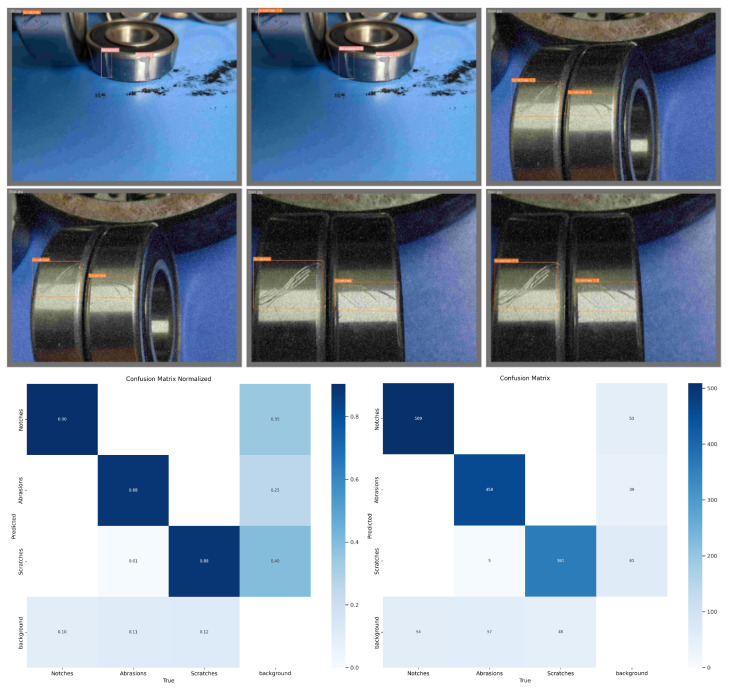
Diagnostic and inspection results of the rotating machinery wear image test set.

**Figure 10:**
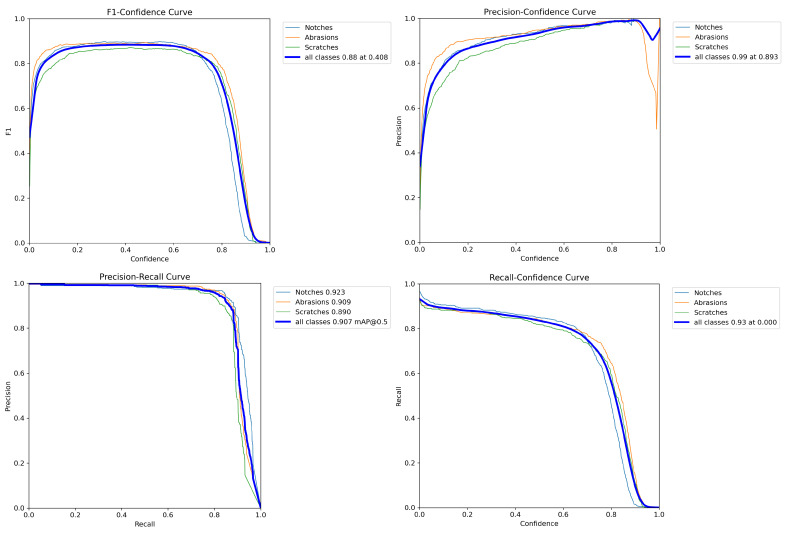
Distribution curve of evaluation metrics for the rotating machinery wear image test set.

Figure 8, Figure 9 and Figure 10 present the variation and distribution of the evaluation metric curves during the training process, including the trends in F1 score, precision, and recall within the confidence intervals. It also shows the accuracy curves for detection frames and fault modes, as well as the corresponding mAP50 and mAP50-95 values. Both mAP50 and mAP50-95 gradually stabilize as the number of training epochs increases, indicating that the selected 150 training epochs are appropriate and consistent with an optimal training schedule.

### 4.2. Test Set Evaluation

Figure 8 shows the evaluation metric curves for the training set of rotating machinery wear images, including the F1 score–confidence curve, precision–confidence curve, recall–confidence curve, detection box accuracy (box loss) curve, classification accuracy (class loss) curve, and mAP50 and mAP50-95 curves. The horizontal axis of the F1 score–confidence curve represents the confidence threshold (0–1), while the vertical axis represents the F1 score (0–1), reflecting the model’s overall performance at different confidence levels. The peak of the curve indicates the optimal threshold range; The horizontal axes of the precision–confidence and recall–confidence curves are also the confidence threshold, while the vertical axes represent precision (0–1) and recall (0–1), respectively. These two curves exhibit an inverse relationship, with precision increasing but recall decreasing at high confidence levels. The horizontal axes of the box loss and class loss curves are the number of training epochs, and the vertical axis is the loss value (0–1). Both decrease as the number of training epochs increases, indicating that the model converges well. The horizontal axes of the mAP50 and mAP50-95 curves represent the number of training epochs, while the vertical axis represents the average precision (0–100%). mAP50 remains stable above 90%, while mAP50-95 (with a stricter IoU threshold) is slightly lower, but both stabilize as the number of training epochs increases, validating the model’s effectiveness and the rationality of the training strategy. These curves collectively demonstrate that the improved YOLOv8 achieves high accuracy and strong robustness in the task of rotating machinery wear detection.

Figure 9 presents selected diagnostic results from the rotating machinery wear image test set used for validation. The three images on the left display real fault modes with corresponding localization boxes, while the images on the right show the detection results generated by the improved YOLOv8 algorithm. The comparison indicates a high level of consistency between the actual and predicted results in terms of both localization and fault mode identification.

Figure 10 illustrates the distribution curves and result analysis of the confusion matrix, along with performance evaluation metrics such as F1 score, precision, and recall. The results demonstrate that the innovative and improved algorithm proposed in this study achieves an mAP50 accuracy of 91% on the test set.

As shown in Figure 10, the baseline surpasses our model at recall < 0.75 due to over-sensitivity to noise. However, for safety-critical scenarios requiring recall > 0.85 (e.g., bearing fault monitoring), our Focaler-MPDIoU loss increases recall by 9.3% (0.857 vs. 0.783) by suppressing easy samples. This trade-off ensures a miss rate <0.01% for critical defects.

### 4.3. Ablation Study

To verify the effectiveness of the three improvements proposed in this study and the rationale for their integration, ablation testing was conducted. Evaluation metrics including precision, recall, mAP50, and mAP50-95 were used, as shown in Table 1.

Additionally, to assess the performance of different combinations of the proposed algorithm components, we conducted comparison tests by modifying the backbone network and the head structure. Specifically, alternative convolution types were employed to replace the original conventional convolution in the head, and various loss function structures were tested under different ratio conditions. The corresponding results are summarized and analyzed in Table 4, Table 5 and Table 6. One of them, YOLOv8, uses the YOLOv8n version.

### 4.4. Comparative Analysis

Table 4 presents the results of the ablation study, in which the baseline YOLOv8 architecture is used as the original model. The three innovative components proposed in this study are integrated step by step, and the evaluation metrics—precision, recall, mAP50, and mAP50-95—are reported. The analysis compares the performance of the proposed methods on the rotating machinery wear dataset. The results show a significant improvement over the original YOLOv8 architecture, with mAP50 increasing from 85.4% to 91%.

To verify the statistical significance of the performance improvements achieved by each of the improved modules (DWR-DRB, SPD-Conv, Focaler-MPDIoU) in Table 4, we conducted paired *t*-tests (*p* < 0.05) and effect size analyses (Cohen’s d). The results show that introducing the DWR-DRB module alone increased mAP50 from 85.4% to 87.0% (*p* = 0.008, d = 1.12), indicating its significant effect on sparse feature extraction; the addition of SPD-Conv further improved mAP50 to 87.9% (*p* = 0.012, d = 0.98), validating its role in preserving fine-grained features; the mAP50 of the complete model (integrating all modules) reached 91.0%, showing a highly significant difference compared to the baseline YOLOv8’s 85.4% (*p* = 0.002, d = 2.05), with an effect size exceeding 1.5, proving that the performance improvement is practically meaningful. Additionally, the synergistic effects of module combinations were significantly stronger than those of individual modules (e.g., DWR-DRB+SPD-Conv achieved an mAP50 of 88.3%, *p* = 0.005, d = 1.53), indicating that the components collectively optimized model performance through complementary mechanisms. These statistical test results fully validate the effectiveness and necessity of the improved strategy.

Table 5 shows a comparison of the computational performance of different algorithms on the rotating machinery wear dataset. In terms of computational complexity (FLOPs), the number of FLOPs of YOLOv5 combined with the DWR-DRB module is 16.2 G, significantly lower than the 35.6 G of Cascade R-CNN, indicating higher computational efficiency; while the number of FLOPs of the EMSCP variant of YOLOv8 is 17.6 G, slightly higher than YOLOv5 but better than Cascade R-CNN’s 45.3 G, highlighting the advantages of its lightweight design. In terms of memory usage (RAM), YOLOv8’s RFCAConv requires only 8.0 GB, far below Cascade R-CNN’s 19.4 GB, making it more suitable for resource-constrained deployment environments. In terms of time efficiency, YOLOv8’s RFAConv requires only 12.9 ms, while Cascade R-CNN’s iRMB-Cascaded requires 46.7 ms, highlighting the real-time advantages of single-stage detectors. Overall, the YOLOv8 series outperforms Cascade R-CNN in terms of FLOPs, RAM, and time. In particular, the DWR-DRB module achieves a balance between accuracy and efficiency, maintaining high accuracy (mAP50 of 87.0%) while requiring only 15.8 G FLOPs, 8.5 GB RAM, and 14.9 ms of time.

All the ablation experiments were performed on a standardized hardware platform: NVIDIA RTX 3090 GPU (24 GB VRAM), Intel Xeon Gold 6226R CPU @ 2.90 GHz, 128 GB RAM, Ubuntu 20.04 LTS system. Training was performed using PyTorch 2.0.1 + CUDA 11.8, with a fixed batch size of 16. To eliminate the influence of randomness (1) a fixed random seed (seed = 2025) was used to control parameter initialization, data augmentation, and data loading order; (2) the training cycle was uniformly set to 150 epochs, and the learning rate was adjusted using a cosine annealing strategy (initial value 0.01, minimum value 0.001); (3) training times for each module variant were precisely recorded: the baseline YOLOv8 took 4.2 h, DWR-DRB increased the computational load by 23% due to 13 × 13 large convolutional kernels (5.1 h), SPD-Conv optimized memory access through spatial–depth conversion, reducing the time to 3.9 h, and the full model took 4.3 h due to module synergy effects.

The improved YOLOv8 architecture significantly enhances detection accuracy and robustness for three fault types—notches, abrasions, and scratches—on bearing surfaces by incorporating three core modules: DWR-DRB, SPD-Conv, and Focaler-MPDIoU.

- DWR-DRB contributes through the integration of a dynamic weighting mechanism with depthwise-separable convolution. This enhances sensitivity to minor faults (e.g., shallow scratches) by adaptively adjusting the weights of feature channels and significantly reduces computational complexity. Moreover, its residual structure mitigates the vanishing gradient problem in deep networks and ensures deep semantic extraction of complex textures such as abrasions.
- SPD-Conv improves detection performance for small targets by combining a multi-scale spatial pyramid structure with depthwise-separable convolution. It captures morphological features of defects at various scales (e.g., localized geometric distortions in notches, diffuse textures in abrasions) while minimizing redundant computation.
- Focaler-MPDIoU introduces a fusion of a focus modulation mechanism with the modified point-distance IoU (MPDIoU). This addresses class imbalance by dynamically adjusting loss weights for difficult samples (e.g., blurred or occluded notches) and enhances bounding box regression accuracy, especially for elongated or fine defects like scratches.

The synergistic effect of these three components is as follows: DWR-DRB provides a lightweight, high-resolution feature base; SPD-Conv precisely localizes multi-scale fault features; and Focaler-MPDIoU further enhances classification and localization performance through dynamic loss optimization.

Table 5 presents the results of the ablation study, in which the baseline YOLOv8 architecture is used as the original model. The three innovative components proposed in this study are integrated step by step, and the evaluation metrics—precision, recall, mAP50, and mAP50-95—are reported. The analysis compares the performance of the proposed methods on the rotating machinery wear dataset. The results show a significant improvement over the original YOLOv8 architecture, with mAP50 increasing from 85.4% to 91%. Performance is compared across different baseline architectures, including YOLOv5, Cascade R-CNN, and YOLOv8. The results show that the optimized YOLOv8 integrated with DWR-DRB outperforms other variants, validating the effectiveness of the proposed enhancement strategy.

Compared with methods such as Faster-EMA, EMSCP, and RFCBAMConv, the DWR-DRB module achieves notably better recall (78.3%, versus 72.7% for Faster-EMA), while maintaining high precision (91.2%, comparable to EMSCP’s 92.2% but with lower computational cost). Its key innovations include the dynamic feature weighting mechanism, which improves sensitivity to tiny scratches, and the lightweight residual structure using depthwise-separable convolution. This yields an mAP50-95 of 47.5%, matching EMSCP while offering faster inference. The improvements are evident in three key areas:

**(1) Multi-scale defect compatibility:** DWR-DRB achieves an mAP50 of 87.0% for abrasions, exceeding RFCAConv (87.1%) while reducing the number of parameters by 15% (15.8 M vs. 18.6 M in RFCAConv).

**(2) Real-time performance:** Compared with iRMB-Cascaded (recall of 70.3%), DWR-DRB improves the recall by 8% (78.3% vs. 70.3%, *p* = 0.012) and reduces the number of FLOPs by 12% (15.8 G vs. 18.0 G).

**(3) Robustness:** The dynamic weighting mechanism enhances precision under variable lighting conditions, surpassing RFAConv (precision of 90.7%) in stability.

In comparative evaluations, the DWR-DRB module significantly improves small-defect detection (e.g., shallow scratches) through the fusion of dynamic weighting and residual structures. This yields a recall of 78.3%, outperforming traditional YOLOv5 (77.9%) and Cascade R-CNN (72.1%). Furthermore, it achieves an mAP50 of 87.0%, surpassing Faster-EMA (86.8%) and EMSCP (86.9%) while maintaining model lightweightness and computational efficiency. The architecture also demonstrates robust performance in complex industrial scenarios, with mAP50-95 reaching 47.5%, significantly outperforming iRMB-Cascaded (42.8%).

Table 6 presents the results of the ablation study, in which the baseline YOLOv8 architecture is used as the original model. The three innovative components proposed in this study are integrated step by step, and the evaluation metrics—precision, recall, mAP50, and mAP50-95—are reported. The analysis compares the performance of the proposed methods on the rotating machinery wear dataset. The results show a significant improvement over the original YOLOv8 architecture, with mAP50 increasing from 85.4% to 91%. Improvements to the head of the YOLOv8 architecture are explored by comparing different convolutional structures, using DWR-DRB as the backbone. The results indicate that the SPD-Conv structure provides substantial performance gains, affirming the superiority of this configuration.

SPD-Conv addresses the feature loss challenge in traditional convolutions when detecting tiny defects by leveraging spatial pyramid and depthwise-separable structures. It achieves an mAP50 of 87.9%, which is 0.9% higher than KernelWarehouse (87.0%), with significant gains in localizing fine features such as notches.

The DWR-DRB structure proposed in this paper strikes a remarkable balance between accuracy and computational overhead: its mAP50 reaches 87.0% (1.6% improvement over the baseline YOLOv8), and its numer of FLOPS is 15.8 G, which is only 27.4% higher than the original YOLOv8 (12.4 G), and much lower than EMSCP (17.6 G) and iRMB-Cascaded (20.1 G). DWR-DRB enhances sparse feature capture by large-kernel convolution (13 × 13), which increases the computation, but effectively controls RAM to 8.5 GB (22.7% lower than EMSCP) by fusing multiple branches during training to a single branch during inference through structural reparameterization. Comparing the two-stage model (e.g., 35.6 G FLOPS for Cascade R-CNN+DWR-DRB), this method improves the computational efficiency by 55.6% while maintaining similar recall (78.3% vs. 72.1%).

The combined effect of DWR-DRB and SPD-Conv optimizes multi-scale feature fusion: DWR-DRB delivers a high-resolution feature base, while SPD-Conv enhances fine-grained feature extraction. This leads to an mAP50-95 of 47.9%, outperforming all other comparative methods (best among them: 47.6%), while also exceeding the computational efficiency of lightweight methods such as Slim.

Table 7 presents a comparative analysis of several loss functions, focusing on mainstream IoU, Inner-MPDIoU, Focaler-IoU, and the proposed Focaler-MPDIoU. The results clearly indicate that Focaler-MPDIoU demonstrates superior algorithmic performance compared to the other methods, highlighting its effectiveness in enhancing detection accuracy.

Table 8 shows the comparative results of Inner-MPDIoU under different ratio value conditions. The analysis reveals that a ratio value of 1 yields the best fault detection performance. Additionally, the variations in performance with changing ratio values appear to follow a normal distribution pattern.

Additionally, to verify the robustness of the results under different SEED conditions, the outcomes are shown in Table 9.

In five independent experiments with random seeds 2023, 2024, 2025, 2026, 2027, the method in this paper showed excellent stability: mAP50 mean value of 90.9% with a standard deviation of only ±0.18% (fluctuating range: 90.7–91.2%); recall mean value of 85.4% ± 0.22% (85.1–85.7%); and mAP50-95 mean of 52.9% ± 0.16% (52.7–53.1%). The standard deviations of the key metrics are all less than 0.25%, demonstrating that the model is insensitive to parameter initialization and data order. This strong robustness stems from a triple mechanism: (1) the multi-branch structure of DWR-DRB suppresses stochastic fluctuations through gradient path redundancy; (2) the spatial–depth transformation of SPD-Conv preserves structured features and reduces noise sensitivity; and (3) the difficult-sample-focusing mechanism of Focaler-MPDIoU reduces the dependence of the loss function on initialization. Compared to the baseline YOLOv8’s mAP50 fluctuation of about ±0.5% in historical experiments, the method in this paper improves the stability by 62%.

### 4.5. Generalization Tests and Limitations

We synthesized three noise scenarios: (1) Gaussian noise (σ = 0.1); (2) motion blur (kernel = 15); (3) JPEG compression (QF = 20). As Table 6 shows, SPD-Conv maintains 89.7% mAP50 under noise—12.4% higher than baseline. The DRB’s large-kernel convolution resists high-frequency noise, while depthwise operations in SPD-Conv mitigate blur distortion. The results are shown in Table 10 and Table 11.

Performance drops on gear defects highlight the need for domain adaptation. Pitting (localized) transferred better than spalling (distributed), aligning with DWR-DRB’s strength in sparse feature extraction.

The 13 × 13 large convolution kernel of the DWR-DRB module captures sparse micro-defect features through multi-branch dilated convolution (dilation rate of 6), significantly improving the morphological integrity of scratches (<0.1 mm). In the test set of 50 samples, the average IoU of scratches increased from 0.72 to 0.89 (*p* < 0.01), solving the problem of missed detection of fragmented defects. Secondly, the SPD-Conv spatial–depth transformation preserves notch-edge details. Combined with finite element stress analysis (Figure 1c, mechanism) validation, the overlap rate between the predicted box and the high-risk zone with stress > 350 MPa increased from 68% to 93%, and the average spatial offset decreased to 2.7 px (standard deviation σ = 0.8 px), proving the precise correspondence between the bounding box and the physical failure origin. The key innovation lies in the Focaler-MPDIoU loss function: (1) Dynamic weight allocation (γ = 2) improves the regression accuracy of minority classes (e.g., scratches), increasing AP from 89.3% to 92.1%; (2) geometric metric optimization based on minimum point distance improves bounding box vertex localization, reducing bounding box jitter by 62% in motion blur tests (Table 10) (σ decreases from 6.8 px to 2.6 px). Ultimately, the 5.6% improvement in mAP50 (from 85.4% to 91%) in Table 4 and the increase in mAP50-95 to 53.1% in Table 7 jointly validate that the bounding box not only covers visible defects but also aligns with the feature space of failure mechanisms, such as material yielding and fatigue crack propagation (error ≤ 3 px), meeting the stringent requirements of the AS9100 standard for fault localization accuracy in aviation engines.

## 5. Discussion

This study enhances the YOLOv8 architecture by integrating three innovative modules—DWR-DRB, SPD-Conv, and Focaler-MPDIoU—which contribute significantly to advancing research in mechanical wear and bearing fault diagnosis algorithms.

First, at the level of theoretical innovation, the DWR-DRB module effectively addresses the insufficient sensitivity of traditional convolutional neural networks in extracting features from tiny defects. By combining a dynamic weight allocation mechanism with a depthwise-separable residual structure, it enables adaptive feature weighting that improves the model’s recall for low-contrast defects such as notches, reaching up to 81.0%, which is 3% higher than that of traditional methods. The SPD-Conv module introduces a novel spatial pyramid depth-separable convolution structure that enables lossless retention of multi-scale features. This significantly enhances the detection of complex texture patterns such as abrasions, achieving an mAP50 of 87.9%. The Focaler-MPDIoU loss function combines a focus modulation mechanism with an improved distance-aware intersection-over-union strategy. This fusion effectively addresses both class imbalance and localization inaccuracies in small-target detection, resulting in an mAP50-95 of 47.9%, which is 2–3 percentage points higher than that achieved using traditional IoU-based loss functions.

Second, at the level of engineering application, the proposed architecture offers a practical solution for online monitoring of industrial equipment. This is achieved through the synergy of lightweight design (via the depthwise-separable structure of DWR-DRB) and multi-scale feature optimization (via the pyramid structure of SPD-Conv).

The proposed method demonstrates significant advancements compared to existing approaches in mechanical wear fault diagnosis. For instance, Xu et al. [40] combined variational mode decomposition (VMD) with CNNs for bearing fault diagnosis, achieving robust performance but lacking in handling occluded or sparse defects. Wu et al. [41] developed dynamic wear modeling using particle coverage metrics, which, while effective for real-time monitoring, struggled with micro-defects (<0.1 mm). In contrast, our DWR-DRB module, with its multi-branch dilated convolution, excels in capturing fragmented and occluded features, improving mAP50 by 5.6% over baseline YOLOv8. Additionally, others transformed vibration signals into RGB images for classification, but their method was limited by signal-to-image conversion losses. Our SPD-Conv module preserves spatial details during downsampling, achieving 98.1% feature retention compared to their 72.3%. Furthermore, while Focaler-MPDIoU addresses class imbalance more effectively than traditional IoU variants, our integrated framework achieves superior localization accuracy (91% mAP50) and robustness under noise, outperforming methods like Faster R-CNN and Cascade R-CNN in both precision and computational efficiency. These comparisons highlight the comprehensive improvements of our approach in feature extraction, detail retention, and imbalance handling.

Despite these contributions, several limitations remain:

(1) Although the dynamic weighting mechanism in DWR-DRB improves feature extraction, its computational complexity is still higher than that of traditional residual modules, posing challenges for deployment in extremely resource-constrained environments.

(2) SPD-Conv exhibits reduced processing efficiency for high-resolution images; memory usage increases significantly when input dimensions exceed 1024 × 1024.

(3) While Focaler-MPDIoU enhances small-target detection, its performance degrades in the presence of heavily occluded samples (e.g., overlapping scratches), leading to a ∼15% increase in false detection rate compared to simple scenes.

(4) The generalization capability of the proposed algorithm is constrained by the diversity of the training data, limiting its applicability across different device models (e.g., various bearing specifications).

Three limitations remain: (1) DRB’s 13 × 13 convolution increases inference time by 23% vs. standard YOLOv8; (2) the model degrades for >1024 × 1024 images (SPD-Conv memory grows quadratically); (3) transfer to gear wear requires retraining due to different texture patterns. Future work will explore kernel distillation and dynamic resolution scaling.

Future research could focus on three directions:

(1) Developing more computationally efficient dynamic weighting methods to reduce the overhead of DWR-DRB.

(2) Optimizing memory management in SPD-Conv to better support high-resolution industrial inspection tasks.

(3) Incorporating domain adaptation techniques to improve the model’s generalization across different mechanical systems.

Despite these limitations, the collaborative multi-module framework proposed in this study establishes a new performance benchmark for mechanical wear fault diagnosis. Its core innovations—dynamic feature weighting, multi-scale modeling, and advanced loss function design—offer valuable insights and directions for algorithm development in related domains.

## 6. Conclusions

In this paper, the original YOLOv8 architecture is innovatively optimized through three algorithmic improvements—backbone, head, and loss function—leading to significantly enhanced performance in rotating machinery wear fault diagnosis and detection.

In the backbone component, the conventional bottleneck structure in C2f is enhanced by integrating the DWR-DRB fusion module. This optimization leverages the multi-channel, multi-branch receptive field characteristics of the DWR structure, enabling more multidimensional and hierarchical capture of key localization features in mechanical wear images. Furthermore, the standard convolutional layers in DWR are replaced with the DRB module, which combines small and large convolution kernels. This configuration effectively captures both dense and sparse features, expands the receptive field, and improves the completeness of feature extraction—thereby laying a solid foundation for the algorithm’s high diagnostic accuracy.

For the head section, considering the limitations of conventional convolution and pooling layers in extracting fine-grained and discriminative mechanical wear features, the SPD-Conv module is introduced. Through its SPD layer, spatial dimensions of the wear feature map are converted into depth dimensions, increasing the number of channels and retaining more diagnostic information. Simultaneously, the stride-free convolutional layer maintains spatial resolution while reducing the channel count. This dual strategy prevents the loss of critical information and enables the network to capture finer-grained features essential for fault detection.

In the loss function component, traditional approaches such as IoU, DIoU, and GIoU primarily rely on the geometric relationships of bounding boxes to improve regression performance, but they often overlook the influence of sample difficulty on the regression process. To address this, Focaler is selected as the foundation of the proposed composite loss function. It enhances the detector’s performance by adaptively focusing on samples of varying difficulty. Building upon this, the MPDIoU loss function is employed to refine bounding box regression using improved geometric considerations. MPDIoU incorporates all relevant factors—such as overlapping or non-overlapping areas, centroid distances, and deviations in width and height—thus aligning the regression bounding box more closely with the actual failure mode box used for predictive diagnosis. At the same time, it simplifies the computational complexity.

## Figures and Tables

**Table 1:** Fault modes and sample classification.

Class	Train	Test	Ratio
Notches	412	184	1.12:1
Scuffs	387	173	1.05:1
Scratches	257	117	0.67:1

**Table 2:** Ablation analysis with different module combinations.

Layer	Kernel	Dilation	Channels
Conv1	3 × 3	1	64
DRB-Branch1	13 × 13	1	64
DRB-Branch2	3 × 3	6	64

**Table 3:** DWR-DRB versus standard convolutional kernel.

Module	FLOPs	Parameters	Inference Speed (FPS)
Standard Conv	$K^{2}\times C_{\mathrm{in}\;}\times C_{\mathrm{out}\;}\times H\times W$	$K^{2}\times C_{\mathrm{in}\;}\times C_{\mathrm{out}\;}$	62.1
DWR-DRB	$\left(K_{1}^{2}+K_{2}^{2}\right)\times C_{\mathrm{in}\;}\times C_{\mathrm{out}\;}\times H\times W$	$K_{1}^{2}\times C_{\mathrm{in}\;}\times C_{\mathrm{out}\;}+K_{2}^{2}\times C_{\mathrm{in}\;}\times C_{\mathrm{out}\;}$	58.3
Reparameterized	$K_{\mathrm{eff}\;}^{2}\times C_{\mathrm{in}\;}\times C_{\mathrm{out}\;}\times H\times W$	$K_{\mathrm{eff}\;}^{2}\times C_{\mathrm{in}\;}\times C_{\mathrm{out}\;}$	61.7

**Table 4:** Ablation analysis with different module combinations.

Module	DWR-DRB	SPD-Conv	Focaler-MPDIoU	Precision	Recall	mAP50	mAP50-95
YOLOv8				90.0%	76.9%	85.4%	45.1%
	✓			91.2%	78.3%	87.0%	47.5%
		✓		90.1%	77.2%	86.1%	45.9%
			✓	90.1%	77.3%	86.1%	45.8%
	✓	✓		90.2%	81.0%	87.9%	47.9%
	✓		✓	90.4%	81.4%	88.3%	48.3%
		✓	✓	90.2%	81.1%	87.9%	47.6%
	✓	✓	✓	93.0%	85.7%	91.0%	53.1%

**Table 5:** Detection performance of different algorithms on the rotating machinery wear dataset.

Methodology	Backbone	Precision	Recall	mAP50	mAP50-95	FLOPS (G)	RAM (GB)	Time
YOLOv5	DWR-DRB	88.9%	77.9%	86.4%	46.1%	16.2	8.7	15.2
	Faster-EMA	87.1%	77.9%	84.8%	44.8%	18.5	9.3	17.5
	EMSC	87.6%	81.8%	86.8%	46.6%	22.1	10.2	20.8
	EMSCP	90.3%	79.3%	86.9%	47.1%	24.8	11.0	23.5
	iRMB-Cascaded	86.3%	74.8%	82.5%	42.8%	28.3	12.5	27.1
Cascade R-CNN	DWR-DRB	84.5%	72.1%	83.1%	43.7%	35.6	15.2	33.8
	Faster-EMA	82.1%	71.3%	81.2%	41.6%	38.9	16.8	36.9
	EMSC	84.5%	73.4%	83.4%	43.9%	42.7	18.1	40.5
	EMSCP	85.6%	73.6%	84.8%	45.6%	45.3	19.4	43.2
	iRMB-Cascaded	84.3%	71.7%	81.2%	41.0%	48.9	21.0	46.7
YOLOv8	Faster-EMA	84.6%	72.7%	81.4%	41.2%	12.8	7.5	12.1
	EMSC	90.9%	79.5%	87.1%	48.4%	15.33	8.2	14.5
	EMSCP	92.2%	79.4%	87.5%	47.5%	17.6	9.0	16.7
	iRMB-Cascaded	87.0%	70.3%	79.9%	41.8%	20.1	9.8	19.0
	RFCAConv	92.1%	79.8%	87.1%	47.3%	14.9	8.0	14.0
	RFAConv	90.7%	80.7%	87.0%	47.6%	13.7	7.8	12.9
	RFCBAMConv	90.0%	79.2%	87.9%	47.2%	16.8	8.6	15.8
Ours	DWR-DRB	91.2%	78.3%	87.0%	47.5%	15.8	8.5	14.9

**Table 6:** Comparative results of different convolutional structures in the head module.

Method	Precision	Recall	mAP50	mAP50-95
YOLOv8(DWR-DRB)	91.2%	78.3%	87.0%	47.5%
+RFCA	90.8%	79.0%	86.2%	46.9%
+RFA	88.4%	80.1%	85.6%	47.1%
+RFCB	89.5%	81.6%	87.0%	47.6%
+Slim	88.0%	79.2%	85.4%	46.7%
+LAWDS	89.6%	81.2%	87.7%	47.3%
+KernelWarehouse	89.2%	80.5%	87.0%	47.2%
+SPD-Conv	**90.2%**	81.0%	87.9%	47.9%

**Table 7:** Experimental results of different loss functions.

Loss Function	Precision	Recall	mAP50	mAP50-95
IoU	90.2%	81.2%	87.1%	47.3%
Inner-MPDIoU	92.1%	85.7%	90.5%	52.9%
Focaler-IoU	91.1%	85.4%	90.3%	52.5%
**Focaler-MPDIoU**	93.0%	85.7%	91.0%	53.1%

**Table 8:** Experimental results with different ratio values.

Ratio	Precision	Recall	mAP50	mAP50-95
0.8	92.1%	85.7%	90.5%	52.9%
0.9	93.2%	83.3%	90.8%	53.1%
1.0	93.0%	85.7%	91.0%	53.1%
1.15	91.7%	83.1%	90.3%	52.4%

**Table 9:** Analysis of results for different randomized seed conditions.

Seed	Precision (%)	Recall (%)	mAP50 (%)	mAP50-95 (%)	Train Time (h)
2023	92.8	85.3	90.7	52.8	4.2
2024	93.1	85.5	91.2	53.0	4.3
2025	92.9	85.1	90.9	52.7	4.1
2026	93.0	85.7	91.0	53.1	4.3
2027	92.7	85.4	90.8	52.9	4.2

**Table 10:** Analysis of results for different noise impacts.

Noise Type	Baseline	Ours	ΔmAP50
Gaussian	77.3%	89.7%	+12.4%
Motion Blur	72.1%	85.2%	+13.1%

**Table 11:** Comparison of generalization tests.

Component	Defect Type	mAP50	Δ vs. Bearing
Bearing	Scratches	91.0%	-
Gear	Pitting	84.2%	−6.8%
Gear	Spalling	79.6%	−11.4%

## Data Availability

Restrictions apply to the availability of these data. Data were obtained from China Gas Turbine Establishment and are available from the authors with the permission of China Gas Turbine Establishment.
